# Clozapine Toxicity Following Aortic Valve Replacement in the Context of Pleural Effusion: A Case Report

**DOI:** 10.7759/cureus.91545

**Published:** 2025-09-03

**Authors:** Adrian Joseph, Dana N Joseph, Newlyn Joseph

**Affiliations:** 1 Department of Psychiatry, Albany Medical Center, Albany, USA; 2 Geisel School of Medicine, Dartmouth Hitchcock Medical Center, Hanover, USA

**Keywords:** anticoagulation, clozapine toxicity, mechanical aortic valve replacement, pleural effusion, psychotropic medication monitoring

## Abstract

Clozapine toxicity is a potentially life-threatening condition that can result from altered drug metabolism, leading to elevated serum levels and systemic adverse effects. Although it is commonly associated with infections and metabolic changes, its occurrence in the setting of recent cardiac surgery and pleural effusion is not well documented. We present the case of a 42-year-old female with a history of schizoaffective disorder, bipolar disorder, and recent mechanical aortic valve replacement, who developed worsening dyspnea and postoperative pleural effusions. Her hospital course was complicated by supratherapeutic clozapine levels, recurrent pleural effusions requiring thoracentesis and chest tube placement, and anticoagulation management challenges due to the mechanical valve. Despite diuresis and pleural drainage, her symptoms persisted, leading to clozapine discontinuation. After dose adjustments and close monitoring, her respiratory status improved, and serum clozapine levels returned to normal.

## Introduction

Clozapine is an atypical antipsychotic primarily used for treatment-resistant schizophrenia and schizoaffective disorder. Despite its efficacy, clozapine is associated with significant adverse effects, including agranulocytosis, myocarditis, and dose-dependent toxicity, particularly in cases of altered metabolism or drug interactions [1]. A well-documented phenomenon is the fluctuation of serum clozapine levels in response to systemic infections, inflammation, or metabolic changes, which can precipitate toxicity even at previously stable doses [2]. Patients who undergo major surgeries, such as cardiac valve replacements, are at increased risk of metabolic disturbances due to factors including altered drug clearance, fluid shifts, and systemic inflammation [3]. In particular, pleural effusions, common in the postoperative period after cardiac surgery, may contribute to changes in drug distribution and elimination [4]. In addition, anticoagulation management in patients with mechanical valve replacements adds further complexity to medication adjustments, potentially exacerbating complications related to clozapine metabolism.

Here, we describe the case of a 42-year-old female with a history of schizoaffective disorder who developed supratherapeutic clozapine levels and persistent pleural effusions approximately one month after undergoing mechanical aortic valve replacement (AVR).

## Case presentation

A 42-year-old female with a history of schizoaffective disorder, bipolar disorder, hypothyroidism, diabetes mellitus, hypertension, and a recent mechanical AVR approximately seven weeks earlier presented to the ED with progressive shortness of breath and worsening lower extremity swelling. She had previously visited the ED four weeks earlier with similar symptoms but was discharged with plans for outpatient evaluation. Over the following weeks, her dyspnea worsened, prompting her return for further evaluation.

**Figure 1:**
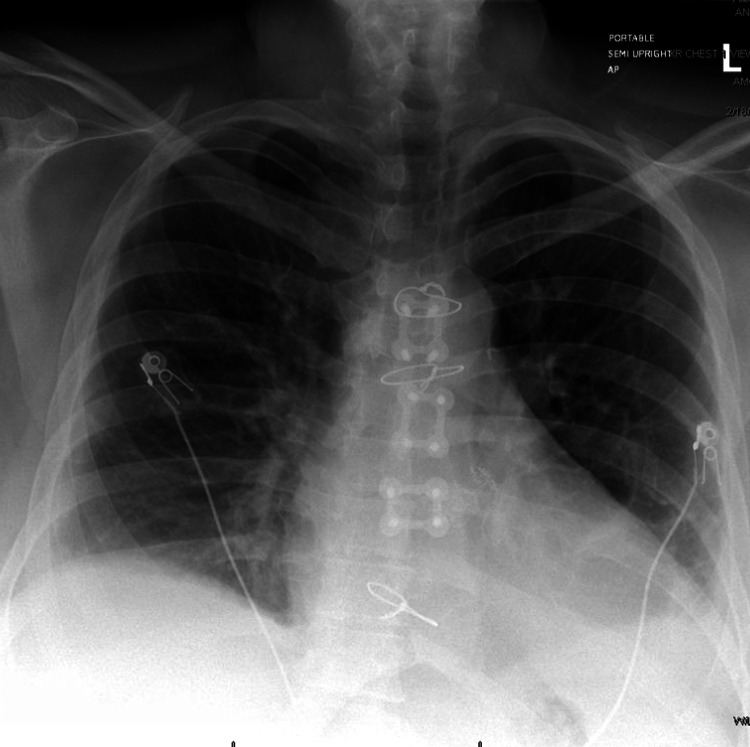
Chest X-ray (portable semi-upright, AP view) The cardiomediastinal silhouette remains unchanged in size and morphology. A prosthetic aortic valve is again noted. Small bilateral pleural effusions are present, with no evidence of pneumothorax. The left pleural effusion has mildly decreased in size compared with the most recent prior exam.

On arrival, vital signs were notable for tachypnea and mild hypoxia. Physical examination revealed diminished breath sounds bilaterally, more pronounced on the left, and pitting edema of the lower extremities. Chest X-ray demonstrated enlarging bilateral pleural effusions, left greater than right (Figure 1). Laboratory studies showed an international normalized ratio (INR) of 9, necessitating immediate vitamin K administration and transition to a heparin drip for therapeutic anticoagulation management in the setting of her mechanical valve. Serum clozapine levels were significantly elevated at 3,046 ng/mL (therapeutic range: 350-600 ng/mL), prompting immediate discontinuation of clozapine upon admission. Notably, an outpatient clozapine level measured eight days prior to admission was even higher at 3,326 ng/mL, suggesting chronic accumulation.

**Figure 2:**
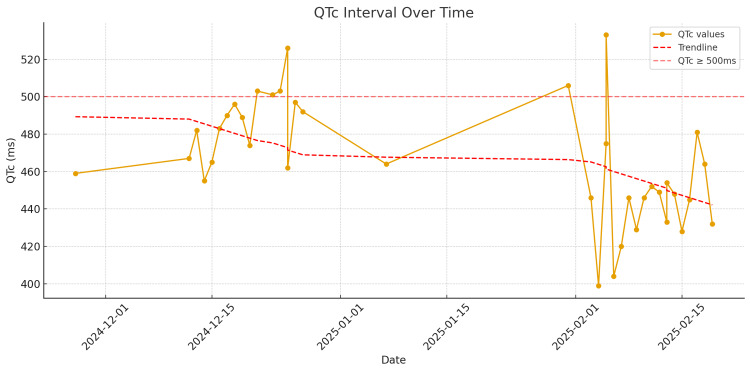
Calculated QTc measured over time The patient’s ECGs were utilized to calculate the QT interval over time.

Her vital signs were as follows: blood pressure 105/66 mmHg, heart rate 85 bpm, respiratory rate 17/min, temperature 35.9 °C, and SpO₂ 92% on 2 L nasal cannula. Her mental status was alert and oriented to person, place, and time, though intermittently anxious. Electrocardiogram monitoring demonstrated a baseline QTc of 459 ms, with subsequent prolongation up to 533 ms during amiodarone therapy, followed by normalization after drug withdrawal (Table 1, Figure 2). Her complete blood count on admission showed hemoglobin 9.0 g/dL, hematocrit 30.4%, WBC 6.6 K/µL, and platelets 333 K/µL. Her metabolic panel was notable for albumin 3.4 g/dL and normal renal function (creatinine 0.65 mg/dL, eGFR 114 mL/min). Appetite and oral intake history were adequate.

**Table 1:** Calculated patient QTc over time The patient’s ECGs were utilized to calculate the QT interval over time.

Date	Time	QTc calculation (Bazett)
11/27/24	12:10	459
12/12/24	4:58	467
12/13/24	5:46	482
12/14/24	5:38	455
12/15/24	4:49	465
12/16/24	5:53	483
12/17/24	4:43	490
12/18/24	7:50	496
12/19/24	4:44	489
12/20/24	5:05	474
12/21/24	5:09	503
12/23/24	9:36	501
12/24/24	9:25	503
12/25/24	5:23	526
12/25/24	14:12	462
12/26/24	4:54	497
12/27/24	4:43	492
01/07/25	13:10	464
01/31/25	13:15	506
02/03/25	12:55	446
02/04/25	5:22	399
02/05/25	5:20	475
02/05/25	9:12	533
02/06/25	4:29	404
02/07/25	5:16	420
02/08/25	5:18	446
02/09/25	13:00	429
02/10/25	4:28	446
02/11/25	4:42	452
02/12/25	8:11	449
02/13/25	5:35	433
02/13/25	11:09	454
02/14/25	5:18	448
02/15/25	6:14	428
02/16/25	5:13	445
02/17/25	8:54	481
02/18/25	8:35	464
02/19/25	5:40	432

Her medication list on admission included clozapine (50 mg AM, 125 mg HS), clonazepam 1 mg TID, divalproex 500 mg BID, levothyroxine 88 mcg daily, furosemide 20 mg daily, nadolol 20 mg daily, warfarin (dosing per INR clinic), and atorvastatin 80 mg nightly, among others.

The following day, the patient underwent bilateral thoracentesis, draining 1.25 L from the left pleural space and 900 mL from the right pleural space. The pleural fluid was serous and non-bloody. The effusions were consistent with volume overload in the post-AVR setting. Bilateral thoracentesis resulted in temporary improvement in respiratory status. However, over the next several days, her pleural effusions rapidly reaccumulated, and her shortness of breath persisted despite diuresis. Thoracic surgery was consulted, and a pigtail chest tube was placed in the left pleural space to facilitate continuous drainage. She was maintained on daily oral furosemide for continued diuresis, and serial imaging showed gradual improvement over the following days.

During hospitalization, the patient developed a short run of ventricular tachycardia, which converted to supraventricular tachycardia with a heart rate of 150 bpm. She was initially started on IV metoprolol, but therapy was discontinued due to hypotension. She was subsequently managed with digoxin and amiodarone; however, both medications were later stopped due to QT prolongation. She was transitioned to nadolol, but five days later developed bradycardia, necessitating a dose reduction to 20 mg once daily.

Given her history of home clozapine use, psychiatry was consulted for reinitiation of therapy. At that time, her serum clozapine level had decreased to 649 ng/mL following discontinuation and medical stabilization. With close psychiatric monitoring, clozapine was gradually reintroduced using an extended titration schedule, beginning at 25 mg twice daily and increasing incrementally over the following 10 days, with plans for continued outpatient titration after discharge.

As her condition stabilized, warfarin was cautiously restarted, and she was maintained on a heparin drip until her INR reached a therapeutic level. By the time of discharge, her INR was 2.0, with a goal range of 2.5-3.5 for her mechanical valve.

She was discharged to her group home in stable condition with instructions to continue clozapine titration under outpatient psychiatric supervision and to remain on daily furosemide for pleural effusion management. A follow-up appointment was scheduled in four weeks to reassess fluid status. Warfarin therapy was continued, and she was scheduled for INR monitoring the day after discharge at the Coumadin clinic. She was also advised to follow up with cardiology for arrhythmia monitoring and heart failure management.

## Discussion

This case provides novel insights compared with prior reports of clozapine toxicity. Most published cases describe acute rises in clozapine levels due to infection, systemic inflammation, or smoking cessation alone. In contrast, our patient developed toxicity in the complex postoperative setting of mechanical AVR with recurrent pleural effusions and anticoagulation instability. The interplay of multiple factors, including recent AVR, perioperative systemic inflammation, cessation of long-term smoking, volume overload with recurrent effusions, and acute initiation of amiodarone, created a unique constellation that amplified the risk of clozapine accumulation.

Clozapine-induced eosinophilic pleural effusion has been reported in the literature as a rare adverse effect, typically associated with peripheral eosinophilia and other signs of drug hypersensitivity [2]. The absence of peripheral eosinophilia in this case makes a clozapine-induced etiology unlikely; therefore, it was not considered a concern. This is among the first reports to highlight postoperative pleural effusion and altered hemodynamics as contributing factors to clozapine toxicity in the absence of infection or eosinophilia. This underscores the importance of recognizing cardiac surgery and fluid balance disturbances as potential precipitants of clozapine toxicity.

Clozapine is predominantly metabolized in the liver via CYP1A2, with minor contributions from CYP3A4 and CYP2C19 [5]. Factors that inhibit CYP1A2 activity can lead to increased serum clozapine levels and toxicity [6]. Acute illness, particularly systemic inflammation, has been well documented to suppress CYP1A2 activity. Inflammatory cytokines such as interleukin-6 (IL-6) can downregulate hepatic enzyme expression, reducing clozapine metabolism and increasing serum concentrations. Given that this patient had pleural effusions and fluid overload, systemic inflammation likely contributed to impaired hepatic metabolism and prolonged drug accumulation.

Another key factor in this case was the patient’s smoking history and recent cessation. She had a 32.3 pack-year history of smoking but had quit three months prior to admission. Tobacco smoke is a potent inducer of CYP1A2, and chronic smokers often require higher doses of clozapine due to increased metabolic clearance [7]. When smoking is discontinued, CYP1A2 activity decreases, leading to reduced clozapine metabolism and increased serum drug concentrations [8]. Given the patient’s long-term smoking history, the residual effects of metabolic downregulation likely contributed to her elevated clozapine levels.

Renal clearance also plays a secondary but important role in eliminating clozapine metabolites. While clozapine itself is predominantly cleared via hepatic metabolism and undergoes significant tubular reabsorption after glomerular filtration, its metabolites are actively secreted by the renal tubules and are cleared at rates several times higher than creatinine clearance [9]. Thus, renal function contributes to the overall clearance of clozapine’s active and inactive metabolites. In this case, the patient’s fluid overload and possible cardiac dysfunction likely resulted in decreased renal perfusion and impaired tubular function, which may have slowed the renal excretion of these metabolites [10]. Although renal elimination accounts for only a minority of total drug clearance, this impairment could have contributed to prolonged systemic accumulation and delayed normalization of serum clozapine levels.

After discontinuing clozapine and performing thoracentesis, her serum levels decreased significantly, confirming the role of fluid overload and systemic inflammation in impaired metabolism. Following stabilization, clozapine was reintroduced with a cautious titration schedule to mitigate the risk of rebound psychosis and other withdrawal effects. The gradual increase over several days allowed for safe reintroduction while avoiding reaccumulation to toxic levels.

This case not only reinforces established risk factors such as smoking cessation and systemic inflammation but also introduces the novel consideration of postoperative pleural effusions and anticoagulation-related hemodynamic shifts as contributors to clozapine toxicity. Clinicians should remain vigilant for drug toxicity in similar postoperative scenarios, even in the absence of typical infectious triggers.

## Conclusions

This case highlights clozapine toxicity in a patient with schizoaffective disorder, recent smoking cessation, systemic inflammation, and fluid overload following AVR, all of which contributed to impaired drug metabolism and clearance. The marked elevation in clozapine levels led to altered mental status and respiratory distress, which improved after withholding the medication and managing the underlying complications. Since clozapine is primarily metabolized by CYP1A2, clinicians should remain mindful of factors that can alter its metabolism, including smoking status, inflammatory states, and changes in volume distribution. This case underscores the importance of therapeutic drug monitoring and timely dose adjustments in patients with acute illness to prevent toxicity while maintaining effective treatment.
